# The Interaction of Diabetes Duration and Tear Film Osmolarity in the Estimation of Tear Film Instability: A Cross-Sectional Study of 60 Patients with Type 2 Diabetes

**DOI:** 10.3390/jcm15166437

**Published:** 2026-08-20

**Authors:** Andreea-Gabriela Schmitzer, Vasile Potop, Liliana Mary Voinea, Speranța Schmitzer, Ana Maria Arghirescu, Andreea Alexandra Mihaela Musat, Maria Cristina Marinescu, Radu Constantin Ciuluvică, Adrian-Iustin Georgevici, Alina-Gabriela Gheorghe

**Affiliations:** 1 Doctoral School, Carol Davila University of Medicine and Pharmacy, 020021 Bucharest, Romania; andreea-gabriela.schmitzer@drd.umfcd.ro (A.-G.S.);; 2 Department of Ophthalmology, Clinical Institute of Ophthalmological Emergencies “Prof. Dr. Mircea Olteanu”, 010464 Bucharest, Romania; 3 Department of Ophthalmology, Carol Davila University of Medicine and Pharmacy, 020021 Bucharest, Romania; 4 Medical Physiology Discipline, Carol Davila University of Medicine and Pharmacy, 020021 Bucharest, Romania; 5 Department of Anatomy, Faculty of Dental Medicine, Carol Davila University of Medicine and Pharmacy, 020021 Bucharest, Romania; 6 Department of Anesthesiology and Intensive Care Medicine, St. Josef Hospital, Ruhr-University Bochum, 44791 Bochum, Germany

**Keywords:** tear film instability, dry eye disease, NIBUT, tear osmolarity, diabetes mellitus, diabetes duration, inflammation

## Abstract

**Background/Objectives:** Dry eye disease is more common in diabetes than in non-diabetic populations (pooled odds ratio 2.30), and its risk rises with diabetes duration. We aim to examine if diabetes duration potentiates the association between tear osmolarity and tear film stability. **Methods:** Cross-sectional study of 60 consecutive adult patients with type 2 diabetes mellitus on stable therapy for ≥3 months, who discontinued artificial tears 1 week before assessment. Worse-eye Non-Invasive-Break-Up-Time (NIBUT) was modelled by Tobit regression with left-censoring at the 1 s instrument floor. We adjusted for body mass index, age, diabetes duration and maximum osmolarity. **Results:** The duration × osmolarity interaction was significantly associated with NIBUT (β = 0.023, 95% CI 0.005–0.040; Wald *p* = 0.010; likelihood-ratio *p* = 0.002). At a normal osmolarity of 290 mOsm/L, expected NIBUT declined from 3.19 s at 5 years to 1.10 s at 20 years; at the 308 mOsm/L cut-point from 2.15 s to 1.35 s; and at an elevated 320 mOsm/L it was essentially unchanged (1.70 s to 1.73 s). **Conclusions:** The association between a longer diabetes duration and a shorter NIBUT is confined to patients whose osmolarity remains normal and is not detectable once hyperosmolarity is established. Instability is already severe in hyperosmolar patients; what attenuates as osmolarity rises is the association with additional duration, not the instability itself. Osmolarity assessment may therefore be most informative in longstanding diabetes with still-normal osmolarity. Given our limited sample size, our results should be interpreted with caution and are hypothesis-generating for future external validation.

## 1. Introduction

Tear film instability is frequently present in dry eye disease in patients with type 2 diabetes mellitus (T2DM). Individual metabolic predictors such as body mass index (BMI), glycated haemoglobin (HbA1c) and triglycerides have each been examined in bivariate association with tear film parameters [1,2,3,4,5]. T2DM produces both vasculopathy and neuropathy, and much of its chronic damage results from several processes acting together rather than from any one of them alone. As in the case of other chronic diseases, we intuitively expect that both pathophysiological severity and the time since onset of the disease should jointly play a role. Our paper focuses on exactly this expectation.

Several metabolic pathways affect the tear film. Diabetic neuropathy has been associated with impaired lacrimal gland function and corneal innervation [4], while dyslipidaemia induces altered meibomian gland secretion [6,7]. Diabetes-related inflammation and oxidative stress combined with a chronic hyperglycaemic state further add a multi-layered complexity to the relationship between diabetes and dry eye disease (DED) [8]. Chronic hyperglycaemia leads to the accumulation of advanced glycation end-products (AGEs), which engage their receptor (RAGE) and activate multiple intracellular pathways. Notably, the nucleotide-binding oligomerization domain leucine-rich repeat and pyrin domain-containing protein 3 (NLRP3) inflammasome. This AGEs–RAGE–NLRP3 axis provides the critical link between hyperglycaemia and chronic ocular surface inflammation in diabetic DED [9].

The activation of the nuclear factor κ-light chain-enhancer of activated B cells (NF-κB) upregulates pro-inflammatory cytokines, whereas activated protein kinase C (PKC) generates reactive oxygen species (ROS) that trigger oxidative stress [9,10]. This dual-signal requirement engages the NLRP3 inflammasome. Concurrently, lipid peroxidation, glucose autoxidation and the polyol pathway further amplify AGE accumulation in diabetic conditions. This self-amplifying inflammatory circuit damages the diabetic ocular surface [5,10,11], promoting fibrosis and progressive functional decline, which lead to reduced tear secretion and tear film instability [12].

Autonomic neuropathy further compounds these effects by impairing lacrimal gland parasympathetic innervation: moderate-to-severe peripheral neuropathy confers a fourfold increase in dry eye risk, corneal nerve fibre density being inversely correlated with tear secretion [4]. The AGEs-RAGE axis affects corneal nerves early on [12], through direct ROS toxicity to the sub-basal corneal nerve plexus [13]. This reduces reflex tearing and blunts the corneal blink reflex. Progressive corneal sensitivity decline with diabetes duration creates a critical diagnostic pitfall: the symptom burden is reduced, despite severe objective clinical findings, resulting in a symptom–sign discordance [13]. This results in masking of significant dysfunction of the lacrimal functional unit (LFU) in these patients [14].

Meibomian gland dysfunction contributes to LFU failure through multiple pathways. AGEs–RAGE-induced inflammation triggers cellular apoptosis and functional impairment, disrupting lipid homeostasis [12]. Corneal neuropathy separately disrupts normal glandular signalling through sensory and autonomic nerve damage [12]. Concurrently, hypertriglyceridaemia alters meibocyte lipid synthesis, promoting ductal obstruction [6,7], in turn accelerating tear evaporation and elevating osmolarity [3]. This suggests that disease chronicity (duration-dependent neuropathy and AGE accumulation) and tear film status (osmolarity) interaction may determine tear instability in a non-additive fashion.

DED under diabetic conditions is a multifactorial entity in which determinants such as age, diabetes duration and metabolic deviations may act jointly rather than independently on the ocular surface. Although these factors have been extensively studied in isolation, their combined contribution has rarely been examined, and a bivariate analysis would be insufficient. A better understanding of this interaction might help identify which patients benefit from earlier ocular-surface assessment [12], and to investigate, in our context, previous papers reporting that glucose-lowering agents such as sodium–glucose cotransporter 2 (SGLT2) inhibitors may be associated with pleiotropic anti-inflammatory effects beyond glucose lowering [15]. Our primary aim is the investigation of Non-Invasive-Break-Up-Time (NIBUT) in a multivariate model including tear film osmolarity, T2DM duration and their interaction.

## 2. Materials and Methods

This cross-sectional study enrolled 60 consecutive adult patients with type 2 diabetes mellitus from a tertiary diabetes centre. The study was approved by the “Carol Davila” University of Medicine and Pharmacy Ethics Subcommittee for Scientific Research (32159/7 November 2025) and conducted in accordance with the Declaration of Helsinki. All participants provided written informed consent.

Inclusion criteria required adults with confirmed T2DM, regardless of staging and stable anti-diabetic therapy for ≥3 months and ability to offer consent and collaborate for all steps of the clinical evaluation. Exclusion criteria included active ocular infection, ocular inflammatory diseases such as uveitis, chronic usage of intraocular pressure (IOP)-lowering drops, history of refractive surgery, recent ocular surgery (cataract or glaucoma surgery, oculoplastic surgery of any kind) (<6 months), systemic autoimmune diseases (systemic lupus erythematosus, rheumatoid arthritis, Sjogren’s syndrome, scleroderma, autoimmune thyroid disease with thyroid eye disease), and contact lens wear. All patients were instructed to discontinue artificial tears 1 week before undergoing dry eye clinical assessment.

All subjects underwent comprehensive dry eye assessment by the same investigator comprising the following: tear osmolarity was measured bilaterally using the TearLab Osmolarity System (TearLab Corp., San Diego, CA, U.S.), under stable environmental condition, before further clinical evaluation using the temporal tear meniscus with the maximum value (worse eye) used for analysis.

Further dry eye assessment was performed using the TearCheck^®^ dry eye screening device (ESW-Vision, Houdan, France, Software Version 4.1), allowing for reproducible and comfortable evaluation of multiple dry eye parameters and tests: blink dynamics represented by abortive blink frequency and blink rate, eye redness and eyelid margin evaluation, patented Tear Film Stability Evaluation (TFSE^®^) and a quality-of-life questionnaire modelled on the validated Ocular Surface Disease Index (OSDI).

NIBUT was evaluated using the same device, by applying the patented TearCheck^®^ Line Mask to create a similar effect to the placido disc lines and was used as the continuous outcome. The instrument reports NIBUT with a lower limit of 1.0 s. Forty-one of 60 worse-eye values (68.3%) were returned as exactly 1.0 s, with no value below it and no observation between 1.0 and 3.2 s in either eye; these values were therefore treated as left-censored at the instrument floor rather than as exact measurements. At the upper end, 21 of 120 eye-level values were recorded at 11.0 s, the longest break-up time observed in this cohort, with the next-highest value being 9.7 s; these represent eyes in which the tear film did not break up within the observation window and were analysed as exact measurements.

Metabolic parameters (HbA1c, fasting lipid panel and fasting glucose levels) were obtained from routine clinical assessment. BMI was measured using weight and height. Both eyes were measured for every ocular parameter. Each of the ocular variables was reduced to a patient-level value by taking its most abnormal value across the two eyes independently: minimum NIBUT and maximum tear osmolarity. We modelled NIBUT (seconds) using Tobit regression with left-censoring at 1.0 s, an approach established for non-normally distributed censored outcomes with covariate adjustment [16], with the following specification:

$${\mathrm{NIBUT}}_{min}=\beta_{0}+\beta_{1}\mathrm{BMI}+\beta_{3}\mathrm{Age}+\beta_{4}{\mathrm{Dur}}_{c}+\beta_{5}{\mathrm{Osm}}_{c}+\beta_{6}\left({\mathrm{Dur}}_{c}\times {\mathrm{Osm}}_{c}\right)+\varepsilon ,$$

where Durc and Osmc are mean-centred (diabetes duration and maximum osmolarity, respectively). Mean-centring ensures that main-effect coefficients represent effects at the cohort mean of the other variable, avoiding uninterpretable extrapolation to zero [17,18]. Diabetes duration and maximum osmolarity were mean-centred at 9.57 years and 318.36 mOsm/L, respectively. Predicted NIBUT was computed with body mass index, worse-eye tear meniscus height and age held at their cohort means (30.71 kg/m^2^, 0.628 mm and 67.2 years). Variance inflation factors (VIFs) were computed to assess multicollinearity. Because the primary model is censored, all reported predictions are expected observed values rather than latent linear predictions.

Two dichotomized secondary analyses complemented the continuous primary model, using the same predictor structure: unpenalized logistic regression at NIBUT ≤ 5 s (47/60, 78.3%), and Firth-penalized logistic regression at the instrument-floor threshold NIBUT ≤ 1 s (41/60, 68.3%). The Tear Film & Ocular Surface Society Dry Eye Workshop II (TFOS DEWS II) tear-film-instability cut-point of <10 s [19] was not used as an outcome because 90.0% of this cohort (54/60) fell below it, leaving only six non-events. Events per variable, calculated on the smaller outcome group, were 2.17 at the 5 s threshold and 3.17 at the 1 s threshold; both dichotomized models are therefore reported for direction only, with inference carried by the continuous censored model.

This study is reported in accordance with TRIPOD as a Type 1b study (model development with internal validation by resampling) [20]. Analyses were performed in R 4.6.0.

Internal validation used leave-one-out cross-validation of the Firth model (60 folds, all converged) and bootstrap optimism correction of an unpenalized logistic model, with calibration slope, calibration intercept and Brier score reported. No a priori sample-size calculation was performed; the available sample was determined by consecutive recruitment over the study period.

## 3. Results

Baseline characteristics are shown in Table 1. In our cohort, mean age was 67.2 ± 8.6 years, BMI 30.7 ± 7.4 kg/m^2^, HbA1c 6.8 ± 1.4%, and diabetes duration 9.6 ± 7.9 years. Median NIBUT was 1.0 s (mean 3.0 ± 3.4) and 41 patients (68.3%) had NIBUT ≤ 1 s.

Applying a tear osmolarity cut-point of ≥308 mOsm/L post hoc, 34/60 patients (56.7%) were classified as hyperosmolar in at least one eye. Because this classification is defined by tear osmolarity itself, no between-group comparison of osmolarity is reported. Worse-eye NIBUT did not differ between hyperosmolar and non-hyperosmolar patients (2.96 ± 3.20 s versus 2.97 ± 3.64 s, Wilcoxon *p* = 0.924).

In the primary censored model (Table 2), the duration × osmolarity interaction was significant (β = 0.0226, SE 0.0087, 95% CI: 0.0055–0.0397, Wald *p* = 0.010; likelihood-ratio *p* = 0.002). Because the interaction terms were mean-centred, the duration main effect represents the effect at the cohort mean worse-eye osmolarity of 318.4 mOsm/L. The positive interaction coefficient indicates that the association between longer diabetes duration and shorter NIBUT was strongest at a lower osmolarity and progressively weaker at a higher osmolarity. Clinically, patients whose osmolarity was still normal showed the steepest duration-dependent NIBUT decline, whereas patients with already-elevated osmolarity began with a severely compromised NIBUT and showed no detectable further decline with duration. All VIFs were below 1.3, indicating no problematic multicollinearity. In the ordinary least-squares sensitivity model (Table 3) the interaction was also significant (β = 0.0049, 95% CI: 0.0008–0.0089, *p* = 0.020; R^2^ = 0.31, adjusted R^2^ = 0.23; F(6, 53) = 3.98, *p* = 0.002), with BMI positively associated with NIBUT (β = 0.152, *p* = 0.007).

**Figure 1:**
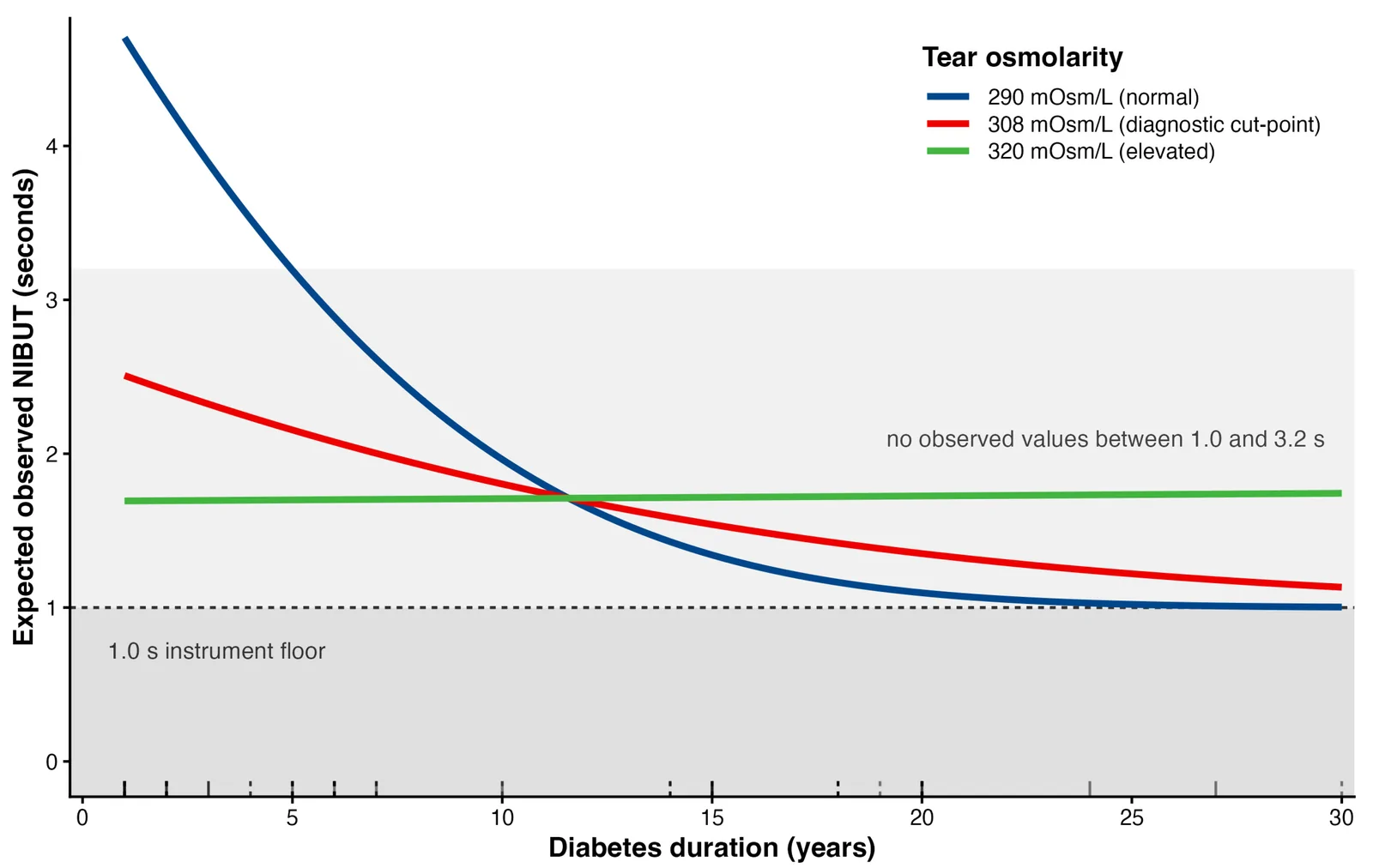
Expected observed Non-Invasive Break-Up Time (NIBUT) across diabetes duration at three levels of tear osmolarity. Curves show expected observed values from the primary Tobit model, left-censored at the 1.0 s TearCheck^®^ instrument floor (*n* = 60, of which 41 censored), with body mass index, worse-eye tear meniscus height and age held at their cohort means (30.71 kg/m^2^, 0.628 mm and 67.2 years). Diabetes duration and worse-eye tear osmolarity were mean-centred at 9.57 years and 318.36 mOsm/L, respectively. The value of 308 mOsm/L is the diagnostic cut-point, not the cohort mean. The darker shaded region lies below the instrument floor; the lighter band spans 1.0 to 3.2 s, an interval within which no eye in this cohort recorded a value, so expected values falling inside it represent model-based interpolation across an unobserved region. Tick marks on the horizontal axis indicate observed diabetes durations.

Expected NIBUT was examined at three osmolarity levels, with all other covariates held at their cohort means (Figure 1). At normal osmolarity (290 mOsm/L), expected NIBUT declined steeply with duration, from 3.19 s at 5 years to 1.10 s at 20 years (Δ = −2.10 s), with the modelled probability of lying at the instrument floor rising from 0.53 to 0.96. At the 308 mOsm/L cut-point the decline was intermediate (2.15 s to 1.35 s; Δ = −0.80 s). At elevated osmolarity (320 mOsm/L) the expected NIBUT was essentially unchanged across the same interval (1.70 s to 1.73 s; Δ = +0.03 s), indicating no detectable duration-related decline once hyperosmolarity is established.

At the NIBUT ≤ 5 s threshold (47/60, prevalence 78.3%), the duration × osmolarity interaction was in the same direction as the primary model (OR = 0.9943, *p* = 0.091) with an apparent, in-sample AUC of 0.858. The marginal significance is consistent with the low events-per-variable ratio at this threshold, where the smaller outcome group contains only 13 observations for six predictors (EPV = 2.17), and does not contradict the primary censored analysis.

Firth-penalized logistic regression of the dichotomized NIBUT ≤ 1 s outcome (41/60 events, 68.3%; EPV = 3.17) supported the interaction (OR = 0.9934, profile-likelihood 95% CI: 0.9853–0.9986, *p* = 0.007). A gamma generalized linear model (GLM) with log-link, which constrains predictions to positive values, also supported the interaction (*p* = 0.009). The interaction was confirmed both in a linear mixed model with a patient-level random intercept (b = 0.0045, SE 0.0019, *p* = 0.019; a between-patient intraclass correlation 0.27); and a generalized estimating equation with an exchangeable working correlation structure and robust standard errors (b = 0.0046, SE 0.0019, *p* = 0.017). Diabetes duration and worse-eye tear osmolarity were uncorrelated in this cohort (Pearson r = 0.005, *p* = 0.972), so the interaction is not attributable to shared variance between the two interacting variables.

## 4. Discussion

In our cohort of 60 consecutive patients with type 2 diabetes, diabetes duration and tear osmolarity are not statistically significantly associated with the NIBUT, however their product (diabetes duration × tear osmolarity) is significantly associated with NIBUT (β = 0.023, 95% CI 0.005 to 0.040; Wald *p* = 0.010; likelihood-ratio *p* = 0.002), estimated in a censored model that accounts for the 1.0-s instrument floor reached by 41 of the 60 patients, thus confirming our main hypothesis. At a normal osmolarity of 290 mOsm/L, expected NIBUT decreased from 3.19 s at 5 years of diabetes to 1.10 s at 20 years. At the diagnostic threshold of 308 mOsm/L, the decrease was smaller, from 2.15 s to 1.35 s. At an elevated osmolarity of 320 mOsm/L, expected NIBUT remained essentially unchanged over the same interval (1.70 s to 1.73 s), indicating no detectable association with diabetes duration. Thus, a longer diabetes duration was associated with greater tear film instability primarily in patients with a normal osmolarity reading. No such association was detectable in patients with established hyperosmolarity.

The novelty of our study fills a certain gap in the literature. Namely, in prior studies the tear film instability was examined using bivariate models, i.e., the covariates were examined independently, without considering interactions [1,2,3,14]. Our expectation was that tear osmolarity (its scaled deviation from the normal values) multiplied by the duration of this chronic disease constitutes the cumulative pathophysiological burden and this product should be associated with tear film instability.

This interaction is best understood within the TFOS DEWS II framework, in which tear hyperosmolarity creates a self-perpetuating circle of instability and inflammation and the aqueous-deficient and evaporative subtypes overlap rather than exclude one another [19,21].

The cornea and lacrimal gland behave as one functional unit in which corneal sensory afferents drive reflex secretion, so sensory loss reduces tear flow and degrades sensation further [22]. Diabetes feeds this loop from several directions: cumulative hyperglycaemia drives advanced glycation end-product deposition with RAGE engagement and NF-κB activation within lacrimal tissue [23]. Corneal sub-basal nerve-fibre loss tracks reduced tear stability and worsens with disease duration, with moderate-to-severe peripheral neuropathy conferring a fourfold dry-eye risk [4]. In addition, the diabetic state withdraws insulin and insulin-like growth factor 1 (IGF-1) support from the ocular surface [24]. These processes plausibly reinforce one another, which may explain why diabetes duration outperforms HbA1c as an index of cumulative ocular-surface exposure [2,25,26].

Regarding the evaporative component of DED, systemic dyslipidaemia perturbs meibum lipid homeostasis, raising its melting point and promoting ductal stasis and accelerated evaporation [6,7], with meibomian gland atrophy tracking serum lipids across populations [3]. An elevated osmolarity reading therefore does not merely co-occur with instability; it signals a tear film whose lipid layer has already failed. This is consistent with the findings in the DREAM study, where, in a cohort of 405 patients with moderate-to-severe dry eye, tear osmolarity explains under 5% of the variability in dry eye signs [27]. As such, it behaves less as a graded correlate of surface damage and more as a marker of the state a tear film has reached.

We interpret the above interactions between multiple pathophysiological pathways thus: while the lipid layer remains intact, break-up time is governed predominantly by the slower neural and trophic processes and declines with diabetes duration. However, once evaporation dominates, break-up time reaches its floor value early and little headroom remains for duration to further affect it. This interpretation should be cautiously seen as only speculative, since our cross-sectional study design is insufficient to determine the aetiology.

Patients with normal osmolarity showed steep, duration-associated NIBUT decline, whereas patients with elevated osmolarity began with severely compromised tear films and showed no detectable further decline with disease duration, consistent with break-up time already lying at the measurement floor. Patients with normal osmolarity and long disease duration may therefore benefit more from closer ocular-surface monitoring.

Several limitations must be stated. The interaction estimate is sensitive to the convention used to reduce bilateral measurements to patient-level values, it is significant under the worse-eye-per-variable and in an eye-level mixed model using all 120 eyes with a patient random intercept, but only marginally under bilateral averaging (*p* = 0.053). Selecting the worst eye’s osmolarity maximizes clinical sensitivity but inflates absolute values and carries more measurement variability, since osmolarity variability rises with its level [28].

As a second limitation, our sample is modest, single-centred and no a priori sample-size calculation was performed. A cross-sectional study is exposed to survivorship (Neyman) bias and to selection/collider effects [29], since long-duration patients who retain normal osmolarity and remain in care may be a constitutionally healthier subset that would itself generate a duration-by-osmolarity pattern. Thirdly, meibography, Schirmer I, tear meniscus height, blink dynamics, ocular redness, eyelid-margin evaluation and the Tear Film Stability Evaluation score were recorded as part of the clinical protocol but are not reported here, and no inference is drawn from them. Collectively these considerations make the findings hypothesis-generating and requiring external validation.

Future directions could include a prospective cohort with NIBUT, osmolarity, meibography and corneal confocal nerve-fibre measurement to test the temporal ordering between the osmolarity and time since onset of diabetes. External validation in larger, ethnically diverse cohorts remains the precondition for any duration-based screening threshold.

## 5. Conclusions

Diabetic dry eye disease is a multifactorial disease in which multiple metabolic pathways converge to disrupt tear film stability and homeostasis, engaging both aqueous-deficient and evaporative components. We show in our cohort that the duration of T2DM multiplied by the tear film osmolarity deviation from normal associates significantly with tear film instability. The novelty of our work resides herein, previous studies examined covariates independently, without considering interaction between multiple factors.

The association between longer diabetes duration and shorter NIBUT was observable in patients whose tear osmolarity remained normal and was not detectable once hyperosmolarity had been established. Tear film instability was already severe in hyperosmolar patients. Tear osmolarity assessment may therefore be particularly beneficial in early diagnosis of patients with longstanding diabetes whose osmolarity is still normal. Our findings are hypothesis-generating, and external validation in larger, independent and ethnically diverse cohorts is required before any screening recommendation can be made.

## Figures and Tables

**Table 1:** Baseline demographic, and metabolic and ocular characteristics of the 60 patients with T2DM.

Characteristics	N = 60
Age (years), Mean ± SD	67.2 ± 8.6
Sex, *n* (%)	
F	34 (57%)
M	26 (43%)
Diabetes duration (years), Mean ± SD	9.6 ± 7.9
HbA1c (%), Mean ± SD	6.8 ± 1.4
BMI (kg/m^2^), Mean ± SD	30.7 ± 7.4
Triglycerides (mg/dL), Mean ± SD	141.8 ± 74.1
Tear osmolarity, bilateral mean (mOsm/L), Mean ± SD	308.4 ± 15.9
Tear osmolarity, worse eye—max(OD,OS) (mOsm/L), Mean ± SD ^a^	318.4 ± 23.3
NIBUT, worse eye—min(OD,OS) (seconds), Mean ± SD ^a^	3.0 ± 3.4
Statin use, *n* (%)	33 (55%)

^a^ Variables used in the regression models. Worse-eye values are the maximum across the two eyes for tear osmolarity and the minimum for tear meniscus height and NIBUT; the contributing eye may therefore differ between variables. Bilateral means are shown alongside them for comparability with prior literature. Continuous variables are reported as mean ± standard deviation and categorical variables as *n* (%). Tear osmolarity is reported twice: as the bilateral mean of the two eyes, for comparability with the prior literature, and as the worse-eye value entered the regression models.

**Table 2:** Tobit regression coefficients for continuous NIBUT (seconds), left-censored at the 1.0 s instrument floor (*n* = 60; 41 censored).

Predictor	β	SE	95% CI Lower	95% CI Upper	*p*-Value
(Intercept)	−40.4482	15.1124	−70.0680	−10.8285	0.007
BMI (kg/m^2^)	0.5035	0.1665	0.1771	0.8299	0.003
Age (years)	0.4448	0.1775	0.0968	0.7927	0.012
Duration, centred (years)	−0.0290	0.1626	−0.3477	0.2897	0.858
Osmolarity, worse eye, centred (mOsm/L)	−0.0455	0.0730	−0.1886	0.0975	0.533
Duration × Osmolarity	0.0226	0.0087	0.0055	0.0397	0.010

Tobit regression of worse-eye NIBUT, left-censored at the 1.0 s instrument floor. *n* = 60, of which 41 (68.3%) censored; residual scale sigma = 5.951; log-likelihood −73.03; likelihood-ratio test for the interaction chi-square = 9.71 on 1 df, *p* = 0.002. Duration and osmolarity are mean-centred at 9.57 years and 318.36 mOsm/L, respectively, so their main effects are the effects at the cohort mean of the other. Coefficients are on the latent scale; expected observed values require the censored-mean transformation given in the Methods. Variance inflation factors were all below 1.3.

**Table 3:** Ordinary least-squares sensitivity model for continuous NIBUT (seconds), reported for comparability with the prior literature and not used for inference.

Predictor	β	SE	95% CI Lower	95% CI Upper	*p*-Value
(Intercept)	−7.3694	3.9653	−15.3227	0.5839	0.069
BMI (kg/m^2^)	0.1520	0.0545	0.0427	0.2614	0.007
Age (years)	0.1208	0.0512	0.0181	0.2235	0.022
Duration, centred (years)	−0.0359	0.0508	−0.1379	0.0660	0.483
Osmolarity, worse eye, centred (mOsm/L)	0.0149	0.0170	−0.0193	0.0490	0.386
Duration × Osmolarity	0.0049	0.0020	0.0008	0.0089	0.020

Ordinary least-squares regression of worse-eye NIBUT on the same predictors as Table 2. *n* = 60; R^2^ = 0.31; adjusted R^2^ = 0.23; F(6, 53) = 3.98, *p* = 0.002. This model is unbounded and therefore returns eight fitted values below the 1.0 s instrument floor, three of them negative (minimum −0.36 s), which is not physically observable under the measurement system. It is reported for comparability with the prior literature and is not used for inference; the censored model of Table 2 is the primary analysis.

## Data Availability

De-identified data and the analysis code are available from the corresponding author on reasonable request.

## Abbreviations

The following abbreviations are used in this manuscript:

NIBUT	Non-Invasive Break-Up Time
CI	Confidence interval
T2DM	Type 2 diabetes mellitus
BMI	Body mass index
HbA1c	Glycated haemoglobin
DED	Dry eye disease
AGEs	Advanced glycation end-products
RAGE	Receptor for advanced glycation end-products
NLRP3	Nucleotide-binding and oligomerization domain, leucine-rich repeat and pyrin domain-containing protein 3
NF-κB	Nuclear factor κ-light chain-enhancer of activated B cells
PKC	Protein kinase C
ROS	Reactive oxygen species
LFU	Lacrimal functional unit
SGLT2	Sodium glucose co-transporter 2
IOP	Intraocular pressure
TFSE^®^	Tear Film Stability Evaluation^®^
OSDI	Ocular Surface Disease Index
Dur	Duration
Osm	Osmolarity
VIFs	Variance inflation factors
TFOS	Tear Film & Ocular Surface Society
DEWS II	Dry Eye Workshop II
TRIPOD	Transparent reporting of a multivariable prediction model for individual prognosis or diagnosis
OR	Odds ratio
EPV	Events per variable
GLM	Generalized linear model
IGF-1	Insulin-like growth factor 1
DREAM	Dry Eye Assessment and Management
